# Presyncope Is Associated with Intensive Care Unit Admission in Emergency Department Patients with Acute Pulmonary Embolism

**DOI:** 10.5811/westjem.2020.2.45028

**Published:** 2020-04-13

**Authors:** David R. Vinson, Darcy C. Engelhart, Disha Bahl, Alisha A. Othieno, Ashley S. Abraham, Jie Huang, Mary E. Reed, William P. Swanson, Victoria A. Clague, Dale M. Cotton, William C. Krauss, Dustin G. Mark

**Affiliations:** * The Permanente Medical Group, Oakland, California; † Kaiser Permanente Division of Research, Oakland, California; ‡ Kaiser Permanente Sacramento Medical Center, Department of Emergency Medicine, Sacramento, California; § University of California, San Diego, La Jolla, California; ¶ St. George’s University, School of Medicine, Grenada, West Indies; || University of California, Davis, School of Medicine, Sacramento, California; # Kaiser Permanente San Diego Medical Center, Department of Emergency Medicine, San Diego, California; ** Kaiser Permanente San Rafael Medical Center, Department of Radiology, San Rafael, California; †† Kaiser Permanente South Sacramento Medical Center, Department of Emergency Medicine, Sacramento, California; ‡‡ Kaiser Permanente Oakland Medical Center, Department of Emergency Medicine, Oakland, California

## Abstract

**Introduction:**

Syncope is common among emergency department (ED) patients with acute pulmonary embolism (PE) and indicates a higher acuity and worse prognosis than in patients without syncope. Whether presyncope carries the same prognostic implications has not been established. We compared incidence of intensive care unit (ICU) admission in three groups of ED PE patients: those with presyncope; syncope; and neither.

**Methods:**

This retrospective cohort study included all adults with acute, objectively confirmed PE in 21 community EDs from January 2013–April 2015. We combined electronic health record extraction with manual chart abstraction. We used chi-square test for univariate comparisons and performed multivariate analysis to evaluate associations between presyncope or syncope and ICU admission from the ED, reported as adjusted odds ratios (aOR) with 95% confidence intervals (CI).

**Results:**

Among 2996 PE patients, 82 (2.7%) had presyncope and 109 (3.6%) had syncope. ICU admission was similar between groups (presyncope 18.3% vs syncope 25.7%) and different than their non-syncope counterparts (either 22.5% vs neither 4.7%; p<0.0001). On multivariate analysis, both presyncope and syncope were independently associated with ICU admission, controlling for demographics, higher-risk PE Severity Index (PESI) class, ventilatory support, proximal clot location, and submassive and massive PE classification: presyncope, aOR 2.79 (95% CI, 1.40, 5.56); syncope, aOR 4.44 (95% CI 2.52, 7.80). These associations were only minimally affected when excluding massive PE from the model. There was no significant interaction between either syncope or presyncope and PESI, submassive or massive classification in predicting ICU admission.

**Conclusion:**

Presyncope appears to carry similar strength of association with ICU admission as syncope in ED patients with acute PE. If this is confirmed, clinicians evaluating patients with acute PE may benefit from including presyncope in their calculus of risk assessment and site-of-care decision-making.

## INTRODUCTION

Patients with acute pulmonary embolism (PE) who present to the emergency department (ED) cross a wide severity spectrum: low-risk patients may be safe for expedited discharge and outpatient management;^1^,^2^ intermediate-risk patients may require more prolonged cardiopulmonary monitoring and inpatient treatment; while high-risk patients may have or develop life-threatening hemodynamic instability and need aggressive care.^3^ Initiating treatments that are tailored to patient needs requires reliable risk stratification,^4^,^5^ a practice that is endorsed by international society guidelines.^6^,^7^

Syncope, a brief, fully reversible transient loss of consciousness and postural tone, is a common symptom among ED patients with acute PE, found in a large systematic review of nearly 22,000 cases to occur in approximately 17% of cases.^8^ The true prevalence of syncope in ED patients with acute PE is unknown, however, and has varied widely by study, ranging from 6.8% to 29.9%.^8^ That review found that unselected acute PE patients with syncope have more severe disease and worse prognosis than their non-syncopal counterparts.^8^ Syncope is associated with higher prevalence of hemodynamic instability, echocardiographic signs of right ventricular dysfunction, as well as a higher risk of 30-day PE-related adverse outcomes and in-hospital or 30-day all-cause mortality. What marginal role syncope plays in assigning PE patients to escalating risk strata is unclear, and only a few prognostic instruments include syncope among their prediction variables.^5^

Presyncope, also known as near-syncope, is the sensation of imminent syncope, but absent a complete loss of consciousness. The prevalence of presyncope in patients with acute PE is unclear. Whether presyncope carries the same implications as syncope in PE patients is unknown, as presyncope has been less commonly studied. Few studies of acute PE identify presyncope in their population,^9^,^10^ and often fail to distinguish presyncopal from syncopal patients in their analysis.^9^ The aforementioned systematic review of syncope and PE makes no mention of presyncope.^8^

Of the 29 studies in the review, only one included presyncopal patients. The world’s largest contemporary registry of PE, the RIETE (Registro Informatizado de Enfermedad TromboEmbólica), includes syncope in its dataset, but not presyncope, and the same is true with other registries.^11^,^12^ The assumption, though, that presyncopal patients should be grouped with syncopal patients for analysis is not altogether unjustified. A large, 11-center prospective study of all-comers (not restricted to PE) with syncope and presyncope in 3581 ED adults ≥60 years of age found that presyncope confers similar risks as syncope for the composite outcome of death or serious clinical event.^13^ Whether PE patients with presyncope and syncope also have similar outcomes has not been established – only 1% of this larger syncopal population was diagnosed with acute PE.

We undertook this secondary analysis of a multicenter, community-based cohort of United States ED patients with objectively confirmed PE to compare presyncopal with syncopal patients in terms of clinical and radiographic characteristics. We hypothesize that presyncope is as important an historical variable as syncope in the evaluation of ED patients with acute PE as both are associated with intensive care unit (ICU) admission, our primary outcome. If this hypothesis is confirmed, clinicians and researchers evaluating patients with acute PE may benefit from including presyncope in their calculus of risk assessment and site-of-care decision-making.

**Population Health Research Capsule**

What do we already know about this issue?

Syncope in patients with acute pulmonary embolism (PE) indicates a worse prognosis than in those without syncope.

What was the research question?

Is presyncope associated with intensive care unit (ICU) admission in emergency department (ED) patients with acute PE?

What was the major finding of the study?

Presyncope, like syncope, is associated with ICU admission in ED patients with PE, even when adjusted for high-risk variables.

How does this improve population health?

Asking patients with acute PE about presyncope and syncope may inform risk assessment and site-of-care decision-making, as these risk factors support hospitalization over home discharge.

## METHODS

### Study Design and Setting

We performed a planned secondary analysis of the MAPLE (Management of Acute PuLmonary Embolism) dataset.^14^–^16^ MAPLE is a retrospective cohort study conducted from January 2013–April 2015 in all 21 community medical centers across Kaiser Permanente (KP) Northern California, a large integrated healthcare system that provides comprehensive medical care for more than four million members with approximately 1.2 million ED visits per year. KP members include at least 33% of the population in areas served and are representative of the demographic and socioeconomic diversity of the surrounding and statewide population.^17^,^18^ Characteristics of the 21 medical centers at the time of the study are reported elsewhere.^14^ This study was approved by the institutional review board of KP Northern California, which granted a waiver of informed consent due to the observational nature of the study.

Care for patients with PE was at the discretion of the treating physicians. The only structured guidance for PE management occurred during the final eight months of the 28-month MAPLE study, during which an electronic clinical decision support tool was implemented at select sites to aid emergency physicians with site-of-care decision-making: home vs hospital. It did not address indications for ICU admission. The tool included presyncope and syncope in a list of relative contraindications to home management. Details of the eSPEED (electronic Support for Pulmonary Embolism Emergency Disposition) controlled pragmatic trial are reported elsewhere.^1^

Indications for ICU admission of PE patients in our EDs are not protocolized. The primary reasons for ICU admission of PE patients are current or anticipated hemodynamic instability or respiratory insufficiency. PE patients are admitted to the ICU also for close monitoring after receiving catheter-directed thrombolytics.

### Selection of Participants, Data Collection and Processing

**Figure 1:**
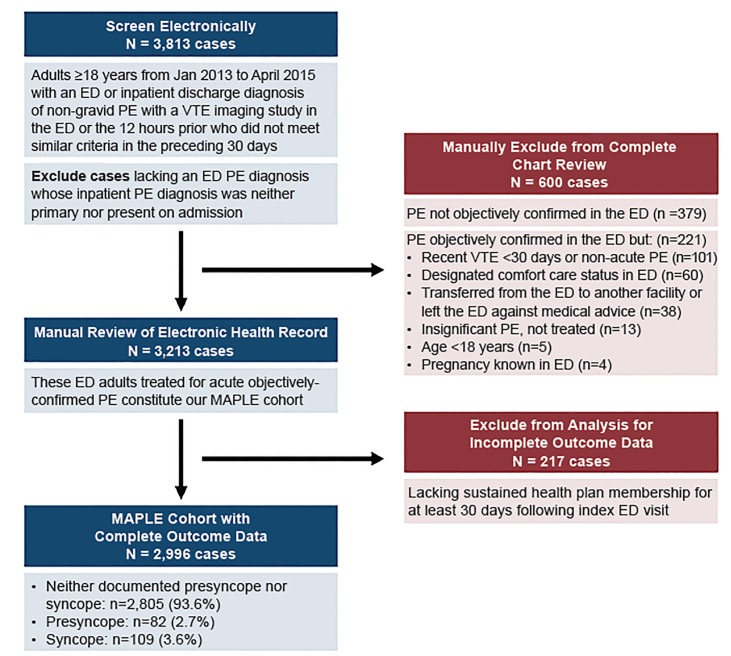
Cohort assembly of emergency department patients with acute pulmonary embolism. *ED*, emergency department; *MAPLE*, Management of Acute PuLmonary Embolism study; *PE*, pulmonary embolism; *VTE*, venous thromboembolism.

We included a consecutive series of adult ED patients ≥18 years of age with acute, objectively confirmed PE diagnosed in the ED or in the preceding 12 hours, as we have described previously.^14^,^15^ We depict the cohort assembly in Figure 1.

We combined electronic health record (EHR) extraction with manual chart abstraction. Thirteen practicing emergency physicians abstracted variables from the EHRs after receiving standardized training on data collection methods and use of the electronic data collection instrument, which was modified to its final form after pilot testing.^14^ A second reviewer abstracted specified variables on a randomly selected subset of 90 cases from the larger MAPLE study of 2996 patients to measure inter-rater reliability, the results of which have been reported elsewhere.^15^ Any case of syncope or presyncope was confirmed by a second abstractor blinded to the initial review, and arbitrated by a third abstractor if necessary.

We defined syncope as an abrupt, transient, and complete loss of consciousness with loss of postural tone, followed by rapid, spontaneous recovery. We defined presyncope as the sensation of imminent syncope, that is, an abrupt, transient feeling of nearly fainting or losing consciousness, followed by rapid, spontaneous recovery as documented by the emergency physician or consultant physician. Non-specific dizziness and light-headedness were not sufficient to constitute presyncope. We provided hypothetical patient quotes to our abstractors to illustrate the study definition of presyncope: “I almost fainted”; “I had to lay down because I felt like I was going to pass out”; “My vision darkened, I got hot and sweaty and nearly lost consciousness.”

We have previously described our calculation of the PE Severity Index (PESI).^19^ Proximal clots involved lobar or main pulmonary arteries.^14^ We classified ED patients as having massive PE who had systolic blood pressure <90 millimeters of mercury sustained over 15 minutes or more, received vasopressors or required cardiopulmonary resuscitation, not caused by new-onset arrhythmia, hypovolemia, or septic shock.^6^,^7^,^20^ We classified ED patients as having submassive PE who did not meet massive PE criteria yet had an elevated ED troponin level (above the 99^th^ percentile), an elevated B-type natriuretic peptide (>100 picograms per milliliter), or right ventricular dysfunction on echocardiogram. Findings of right ventricular enlargement on computed tomography pulmonary angiography (CTPA) were infrequently reported.^21^ Nearly all of the patients with abnormal right ventricular findings on CTPA were also positive for right ventricular strain by the criteria listed above.

The interventions we measured were as follows: ED ventilatory support included non-rebreather mask; positive-pressure ventilation; and mechanical ventilation. Advanced clot treatment included systemic thrombolysis, catheter-directed thrombolysis, and embolectomy.

### Primary and Secondary Outcomes

Our primary outcome was ICU admission from the ED. This included patients who en route from the ED to the ICU passed through the interventional radiology suite for catheter-directed thrombolysis, an infrequent occurrence during the study period. Secondary outcomes included 30-day major hemorrhage, recurrent venous thromboembolism, and all-cause mortality.^22^–^24^ Major hemorrhage was defined by the International Society on Thrombosis and Haemostasis as bleeding at high-risk anatomic locations (intracranial, intraspinal, intraocular, retroperitoneal, intra-articular, pericardial, or intramuscular with compartment syndrome), or overt bleeding with either a reduction of hemoglobin ≥2 grams per deciliter or a transfusion of two or more units of red blood cells.^25^ Recurrent venous thromboembolism was defined as a new or expanded abnormality on imaging. Deaths were identified using a health system mortality database that links to the Social Security Death Master File and the California State Department of Vital Statistics to identify deaths both within and outside of the healthcare delivery system. We also identified out-of-system medical encounters using a comprehensive claims database to improve capture of all healthcare visits related to our 30-day outcomes.

### Data Analysis

We present continuous variables as medians with interquartile ranges (IQR) and categorical data as frequencies and proportions. We compare characteristics between groups (presyncope vs syncope vs neither) using chi-square tests for categorical variables and t-tests or Wilcoxon rank-sum tests for continuous variables. A two-tailed p value of less than 0.05 is considered significant. Covariates that are differentially distributed between the three groups of interest were candidates for inclusion in the predictive models.

We examined adjusted associations of syncope and presyncope with ICU admission using multivariable logistic regression, adjusting for demographics, PESI class, ventilatory support, submassive and massive PE classification with standard errors adjusted for clustering by medical center. We also tested interaction terms between either presyncope and syncope and submassive or massive PE and PESI class. We undertook sensitivity analyses by excluding massive PE from the predictors in the model since the association of sustained hypotension with ICU admission is not in question. We also examined adjusted associations of the above covariates of interest with the secondary outcome of 30-day, all-cause mortality. Adjusted associations are reported as odds ratios (aOR) with 95% confidence intervals (CI). All analyses were conducted using SAS statistical software, version 9.31 (SAS Institute, Cary, NC) and Stata, version 14.2 (StataCorp LP, College Station, TX).

## RESULTS

Throughout the 28-month study, we identified 2996 eligible patient encounters (Figure 1). The median age of the entire cohort was 66 years (IQR 54–77), and 1485 (49.5%) were men. Overall, 82 (2.7%) had presyncope and 109 (3.6%) had syncope documented in the EHR. We report in Table 1 patient demographic, comorbid, and clinical characteristics stratified by the three groups: patients with presyncope; syncope; and neither. Table 2 highlights notable between-group differences in radiographic risk stratification, and management characteristics. The presyncope and syncope patients shared in common many characteristics distinct from their counterparts with neither documented complaint: they more commonly arrived by ambulance, had a proximal clot, and a higher PESI class, submassive, and massive PE classification. They more commonly required ventilatory support and thrombolytics. They were also more commonly hospitalized (Table 2). Overall, 175 patients (5.8%) were admitted to the ICU: 170 from the ED, five of whom passed through interventional radiology for catheter-directed thrombolysis (Table 2). The presyncope and syncope subgroups had similar prevalence of ICU admission: presyncope 18.3% vs syncope 25.7% (compared with 4.7% among those with neither condition).

We report in Table 3 the results of our univariate and multivariate analysis for the primary outcome, ICU admission. We found that presyncope and syncope were both independently associated with ICU admission, as were lower age, higher PESI class, ventilatory support, proximal clot location, and submassive and massive classification. The association of both presyncope and syncope with ICU admission remained when massive PE patients were removed from the analysis and only normotensive patients remained (see the supplemental files, Table S1). There was no significant interaction between either syncope or presyncope and PESI, submassive classification or massive classification in predicting ICU admission (data not shown). Presyncope was thus associated with ICU admission even among normotensive patients and those without markers of right ventricular dysfunction.

Four patients (4.9%) in the presyncope group died of any cause within 30 days and 10 (9.2%) in the syncope group (p = 0.40). In adjusted analysis, higher-risk PESI class (Classes III–V; aOR 22.17; 95% CI, 7.91–62.14), ventilatory support (aOR 3.78; 95% CI 2.77–5.16) and massive PE (aOR 3.69; 95% CI, 1.42–9.59) were independently associated with 30-day all-cause mortality (Table 4). Restricting the analysis to normotensive patients did not meaningfully change these findings (see the supplemental files, Table S2).

## DISCUSSION

In this multicenter, retrospective cohort study of community-based ED patients with acute PE we found a similar prevalence of ICU admission (approximately one in five) among patients with syncope and those with presyncope. Both groups had a four-fold or higher proportion of ICU admission than their counterparts with neither complaint. Presyncope and syncope were each associated with ICU admission, independent of patient demographics, PESI class, ventilatory support, clot location, and submassive and massive classification.

Our finding that both syncopal and presyncopal PE patients are at higher and comparable risk for needing intensive care than non-syncopal patients is concordant with one of the few PE studies to have identified presyncope in their PE population and compared them to their syncopal and non-syncopal patients. Among 1716 patients with PE enrolled in the prospective Italian Pulmonary Embolism Registry, 239 patients (13.9%) reported presyncope and 219 (12.8%) reported syncope.^10^ Their presyncopal patients were similar to the syncopal patients on most measures. Both groups had significantly higher 30-day, all-cause mortality than their non-syncopal counterparts: presyncope (47.2%); syncope (37.4%); neither (6.2%). Direct comparisons between the Italian registry and our own, however, are prevented because of the disparate populations and settings. Their overall 30-day, all-cause mortality was 15.9%, whereas ours was 4.4%, suggesting significant between-site differences in disease severity and management.^1^

Our results are also consistent with more general studies comparing presyncope with syncope patients across a broad spectrum of ED complaints. For example, a prospective, observational study in 3581 older adults (≥60 years) with presyncope or syncope in 11 EDs identified no between-group differences in the composite incidence of 30-day mortality or serious clinical events.^13^ These events included cardiac arrhythmias, myocardial infarction, cardiac intervention, new diagnosis of structural heart disease, stroke, PE, aortic dissection, subarachnoid hemorrhage, cardiopulmonary resuscitation, internal hemorrhage or anemia, and recurrent fall or syncope resulting in major injury. Only 1% of their cohort was diagnosed with acute PE, however, so these investigators cannot speak directly to our particular study population. Nevertheless, their findings suggest that presyncopal ED patients carry similar short-term risks as syncopal ED patients and should be managed in a similar fashion.

Syncope is a known predictor of adverse events in unselected ED patients with acute PE, as it correlates with a higher prevalence of hemodynamic instability and right ventricular dysfunction at the time of PE presentation and confers a higher risk for adverse outcomes.^8^,^26^ But the correlation between syncope and adverse outcomes has disappeared in some studies when patients presenting with hypotension were removed from the cohort, according to the results of a recent 29-study meta-analysis of nearly 22,000 patients with PE.^8^ The authors suggest that hemodynamic instability (rather than syncope itself) may be driving the correlation. Presyncope was not included in the analysis given its near absence from the included studies. However, our study suggests that the increased risk imparted by presyncope or syncope is independent of hemodynamic instability, since our findings were insensitive to removal of patients with massive PE from the analysis. These results suggest that the effect of presyncope and syncope on PE outcomes is not mediated exclusively by hemodynamic instability and carries an independent prognostic role, even in normotensive patients and even when adjusted for other variables known to confer risk of adverse outcomes, such as right ventricular dysfunction.

Pulmonary emboli are thought to cause presyncope and syncope by mechanical, neurohumoral, and tachydysrhythmic mechanisms. Large, central PE can work at the mechanical level, creating direct right ventricle outflow obstruction, which can lead to a downstream reduction in left ventricular filling and cardiac output. Proximal clots are more prevalent among PE patients with presyncope or syncope than those without: we found this in our study (61.7% vs 48.1% [p = 0.001; Table 2]), as have others.^27^–^29^ Nevertheless, about 40% of our presyncope/syncope cohort did not have proximal clots at the time of CTPA. PE, however, is a dynamic condition and some of these patients may have had larger, more central clots earlier that had subsequently undergone partial in vivo fibrinolysis and fragmentation by the time of imaging.^29^ The increase in pulmonary hypertension can manifest with right ventricular dysfunction (and qualify patients for submassive classification). Right ventricular dysfunction was more prevalent in our presyncope/syncope patients compared with their non-syncopal counterparts: 51.8 % vs 29.6% (p<0.0001; Table 2), results that are also consistent with other studies.^8^,^9^,^28^

Smaller and more distal clots lack the mechanical effects of their larger, proximal counterparts, but can still impede cardiac function through biochemically-induced pulmonary vasoconstriction. PE may also cause presyncope and syncope by provoking paroxysmal tachydysrhythmias, as well as by transiently increasing vagal tone.^29^,^30^ These various mechanisms of presyncope and syncope do not each carry the same implications. A transient vagal reaction in a patient with acute PE generally has less prognostic significance than a large, central clot with evidence of persistent right ventricular dysfunction. Given, however, that presyncope and syncope are independently associated with ICU admission, even after controlling for known prognostic factors, including clot location and right ventricular dysfunction, we think it wise to initially hospitalize this population of PE patients for monitoring.

We have already incorporated these results into our ED PE clinical pathway by denoting presyncope an indication for hospitalization. In an electronic clinical decision support application to assist emergency physicians in real-time, site-of-care decision-making, we provide two complementary risk-stratification tools. The first is the PESI, accurately auto-populated from the EHR.^1^,^19^,^31^ The second tool is a memory-jogging list of relative contraindications to immediate outpatient care. Among the PE-related factors, we include presyncope alongside syncope.^1^ The results of this current study, while only suggestive, nevertheless lend weight to considering presyncope a relative contraindication to immediate home discharge among ED patients with acute PE. Likewise, for patients diagnosed with PE in the primary care setting, presyncope and syncope may both be an indication for transfer to a higher level of care.^32^

## LIMITATIONS

The major shortcoming of this study is its retrospective design, which likely underestimates the prevalence of presyncope and syncope. We were able to manually abstract presyncope and syncope from the EHR in a structured fashion and achieved the agreement of two physician abstractors. But not all presyncopal and syncopal events made it to the health record, as patient reporting, physician inquiry, and physician documentation were all subject to incompleteness. Physicians may have been more inclined to inquire and to document presyncope and syncope in patients with more severe manifestations of PE, but how this potential bias, if it exists, may have affected our results is unclear. Nevertheless, this limitation in design attenuates the claims we can make from our results.

Moreover, our primary outcome, ICU admission, was not protocolized, and may have been influenced by many patient-, physician-, and facility-level variables. When attempting to isolate the effect of presyncope and syncope upon ICU admission, we adjusted for multiple high-risk factors, but could not account for excluded and unmeasured variables. Additionally, the results we found reflect the study population and setting and may not be generalizable to other geographic locations, practice settings and case mixes. As expected, the number of patients in the presyncope and syncope subgroups who died was small. Although we found no significant adjusted association between presyncope or syncope and mortality, the low observed incidence and relatively small study cohort limit any conclusions about mortality effects.

While ICU admission as an outcome variable can be construed as too variable (eg, interfacility differences in admission criteria) and too subjective (based on physician judgment), these factors were minimized by the large number of centers (n = 21) and physicians involved. As such, ICU admission represents a valid proxy for tenuous clinical status. The increase in ICU admission among the presyncope/syncope population was not exclusively attributable to hemodynamic status (eg, massive classification), need for ventilatory support, clot location, right ventricular dysfunction, or predicted short-term mortality (PESI classification).

## CONCLUSION

Little attention has been paid to the prognostic significance of presyncope in ambulatory adults with acute PE. This retrospective study suggests that presyncope carries similar risks as syncope in the PE population, an hypothesis supported by the Italian PE registry as well as the broader presyncope literature.^10^,^13^ Prospective studies are needed to quantify the magnitude of the association between presyncope and high-risk clinical characteristics and short-term adverse outcomes, as well as to tease out more clearly the prognostic import of presyncope in the normotensive population and those without right ventricular dysfunction. Meanwhile, it may be sensible to add presyncope to the list of questions we ask of our PE patients when making site-of-care decisions and to consider it a prognostic factor comparable to syncope.

## Supplementary Information



## Figures and Tables

**Table 1:** Demographic and comorbid characteristics of emergency department patients with acute pulmonary embolism stratified by documentation of presyncope and syncope (n = 2996).

Patient characteristics	Neither Presyncope nor Syncope n = 2805 (93.6%)		Presyncope n = 82 (2.7%)		Syncope n = 109 (3.6%)	

Demographics	n	%	n	%	n	%
Age median (IQR), years	66 (54–77)		67.5 (56–75)		68 (56–77)	
Gender male	1390	49.6	34	41.5	61	56.0
Race/ethnicity						
White	2002	71.4	66	80.5	74	67.9
African American	357	12.7	6	7.3	21	19.3
Hispanic or Latino	282	10.1	3	3.7	6	5.5
Asian or Pacific Islanders	127	4.5	2	2.4	8	7.3
Other	37	1.3	5	6.1	0	0.0

Comorbidities						

Obesity (body mass index >30 kg/m^2^)	1267	45.2	46	56.1	46	42.2
Cancer (history of or active)	807	28.8	28	34.2	32	29.4
Chronic lung disease (includes asthma)	762	27.2	19	23.2	29	26.6
Documented history of prior venous thromboembolism	466	16.6	15	18.3	12	11.0
Coronary artery disease	404	14.4	12	14.6	19	17.4
Heart failure (diastolic or systolic)	281	10.0	9	11.0	10	9.2
Cerebrovascular disease	235	8.4	6	7.3	11	10.1
Smoking	167	6.0	7	8.5	4	3.7
Chronic severe renal failure	62	2.2	3	3.7	4	3.7

Charlson Comorbiditiy Index Score						

Mean (SD)	1.90 (2.39)		1.91 (2.36)		2.03 (2.51)	
Median (IQR)	1 (0–3)		1 (0–3)		1 (0–3)	
0	1056	37.7	31	37.8	37	33.9
1	535	19.1	11	13.4	28	25.7
≥2	1154	41.1	36	43.9	42	38.5
No measure (no visits in prior year)	60	2.1	4	4.9	2	1.8

*IQR*, interquartile range; *SD*, standard deviation.

**Table 2:** Clinical characteristics of emergency department patients with acute pulmonary embolism stratified by documentation of presyncope and syncope (n = 2996).

Patient characteristics	Neither Presyncope nor Syncope n = 2805 (93.6%)		Presyncope n = 82 (2.7%)		Syncope n = 109 (3.6%)		P-values comparing three groups

Arrival by ambulance	n	%	n	%	n	%	
No	2264	80.7	48	58.5	40	36.7	<0.0001
Yes	541	19.3	34	41.5	69	63.3	

PE Severity Index class							

I	503	17.9	10	12.2	14	12.8	0.0254
II	615	21.9	13	15.9	14	12.8	
III	547	19.5	19	23.2	22	20.2	
IV	492	17.5	19	23.2	20	18.4	
V	648	23.1	21	25.6	39	35.8	

Ventilatory support*	140	5.0	10	12.2	15	13.8	<0.001

Non-rebreather mask only	79	2.8	9	11.0	11	10.1	
Non-invasive or invasive ventilation	61	2.2	1	1.2	4	3.7	

Clot location on CTPA†							

Proximal	1349	48.1	50	61.0	68	62.4	0.0013
Distal	1195	42.6	21	25.6	33	30.3	
Unclear or not measured	261	9.3	11	13.4	8	7.3	

Massive classification‡							

Neither massive nor submassive	1958	69.8	31	37.8	52	47.7	<0.0001
Submassive	829	29.6	49	59.8	50	45.9	
Massive	18	0.6	2	2.4	7	6.4	

Hospitalization	2184	77.9	77	93.9	105	96.3	<0.0001

To hospital floor	2052	73.2	62	75.6	77	70.6	
To intensive care unit	132	4.7	15	18.3	28	25.7	
Thrombolytics (n=28)§	18	0.6	5	6.1	5	4.6	<0.0001
Intravenous (n=23)	14		4		5		
Catheter-directed (n=5)	4		1		0		

30-day adverse events							

Major hemorrhage	79	2.8	3	3.7	8	7.3	0.0236
Recurrent venous thromboembolism	20	0.7	0	0.0	1	0.9	0.7196
All-cause mortality	115	4.1	4	4.9	10	9.2	0.0364

*CTPA*, computed tomography pulmonary angiography; *ED*, emergency department; *PE*, pulmonary embolism.

* Includes non-rebreather mask, non-invasive ventilation, and endotracheal intubation with mechanical ventilation

† Proximal emboli were clearly lobar or more proximal, whereas distal emboli were “segmental or lobar” or more distal. Location was not measured in patients whose PE was diagnosed with ventilation/perfusion scan.

‡ Massive PE required systolic blood pressure <90 mmHg sustained over 15 minutes or more, reception of vasopressors or cardiopulmonary resuscitation, not caused by new-onset arrhythmia, hypovolemia, or septic shock. Submassive PE did not meet massive PE criteria, yet had an elevated ED troponin level, an elevated B-type natriuretic peptide, or right ventricular dysfunction on echocardiogram.

§ Thrombolytics were administered in the ED or upon arrival (<2h) to interventional radiology or the intensive care unit.

**Table 3:** Association between patient characteristics and intensive care unit admission among emergency department patients with acute pulmonary embolism (n = 2996).

	Univariate models		Multivariate model	

Patient characteristics	Odds ratio	95% CI	Adjusted odds ratio	95% CI
Age, per year	0.99	0.98–1.00	0.94	0.93–0.96
Gender				
Female	reference		reference	
Male	1.06	0.71–1.56	0.99	0.69–1.41
Race/ethnicity				
White	reference		reference	
Non-white	1.19	0.86–1.65	1.04	0.70–1.53

PE Severity Index class				

I	reference		reference	
II	1.74	0.75–4.05	4.27	1.97–9.26
III	1.83	0.78–4.25	3.67	1.64–8.22
IV	2.75	1.29–5.87	7.54	3.32–17.10
V	5.62	2.40–13.15	13.82	5.98–31.94

(Pre)syncope classification				

Neither	reference		reference	
Presyncope	4.53	2.30–8.95	2.79	1.40–5.56
Syncope	7.00	4.36–11.23	4.44	2.52–7.80

Ventilatory support*				

None	reference		reference	
Any	10.51	7.39–14.94	4.06	2.52–6.53

Clot location on CTPA†				

Distal	reference		reference	
Proximal	3.07	2.16–4.37	2.23	1.47–3.38
Unclear or not measured	0.94	0.34–2.58	0.67	0.28–1.58

(Sub)massive classification‡				

Neither	reference		reference	
Submassive	5.03	3.74–6.75	3.32	2.26–4.88
Massive	139.37	56.78–342.10	36.49	12.33–107.98

*CI*, confidence interval; *CTPA*, computed tomography pulmonary angiography; *PE*, pulmonary embolism.

* Includes non-rebreather mask, non-invasive ventilation, and endotracheal intubation with mechanical ventilation.

† Proximal emboli were clearly lobar or more proximal, whereas distal emboli were “segmental or lobar” or more distal. Location was not measured in patients whose PE was diagnosed with ventilation/perfusion scan.

‡ Massive PE required systolic blood pressure <90 mmHg sustained over 15 minutes or more, reception of vasopressors or cardiopulmonary resuscitation, not caused by new-onset arrhythmia, hypovolemia, or septic shock. Submassive PE did not meet massive PE criteria, yet had an elevated ED troponin level, an elevated B-type natriuretic peptide, or right ventricular dysfunction on echocardiogram.

**Table 4:** Associations between patient characteristics and 30-day all-cause mortality among emergency department patients with acute pulmonary embolism (n = 2996).

	Univariate models		Multivariate model	

Patient characteristics	Odds ratio	95% CI	Adjusted odds ratio	95% CI
Age, per year	1.03	1.02–1.04	1.01	0.99–1.02
Gender				
Female	reference		reference	
Male	0.77	0.52–1.15	0.67	0.44–1.02
Race/ethnicity				
White	reference		reference	
Non-white	0.86	0.56–1.32	0.95	0.61–1.47

PE Severity Index class				

I–II	reference		reference	
III–V	10.17	2.61–16.81	22.17	7.91–62.14

(Pre)syncope classification				

Neither	reference		reference	
Presyncope	1.20	0.47–3.03	0.77	0.34–1.75
Syncope	2.36	1.03–5.44	1.41	0.45–4.37

Ventilatory support*				

None	reference		reference	
Any	6.78	5.07–9.07	3.78	2.77–5.16

Clot location on CTPA†				

Distal	reference		reference	
Proximal	1.12	0.82–1.53	0.97	0.69–1.36
Unclear or not measured	1.72	0.94–3.15	1.54	0.80–2.95

(Sub)massive classification‡				

Neither	reference		reference	
Submassive	1.51	1.15–2.00	0.88	0.64–1.23
Massive	16.32	7.46–35.72	3.69	1.42–9.59

*CI*, confidence interval; *CTPA*, computed tomography pulmonary angiography; *PE*, pulmonary embolism.

* Includes non-rebreather mask, non-invasive ventilation, and endotracheal intubation with mechanical ventilation

† Proximal emboli were clearly lobar or more proximal, whereas distal emboli were “segmental or lobar” or more distal. Location was not measured in patients whose PE was diagnosed with ventilation/perfusion scan.

‡ Massive PE required systolic blood pressure <90 millimeters mercury sustained over 15 minutes or more, reception of vasopressors or cardiopulmonary resuscitation, not caused by new-onset arrhythmia, hypovolemia, or septic shock. Submassive PE did not meet massive PE criteria yet had an elevated ED troponin level, an elevated B-type natriuretic peptide, or right ventricular dysfunction on echocardiogram.
