# Supplementary material for: Presyncope Is Associated with Intensive Care Unit Admission in Emergency Department Patients with Acute Pulmonary Embolism
Source: West J Emerg Med. 2020 Apr 13;21(3):703–13. doi: 10.5811/westjem.2020.2.45028 (PMC7234693; doi:10.5811/westjem.2020.2.45028)
Supplement: Supplementary file 1 [file wjem-21-703-s001.docx]

**Supplemental Tables**

**Table S1**. Association between patient characteristics and initial intensive care unit admission of emergency department patients with non-massive acute pulmonary embolism (n=2,969).

|  | Multivariate Model | | |
| --- | --- | --- | --- |
|  | Adjusted Odds Ratio | 95% CI | |
| Age, per year | 0.94 | 0.93 | 0.96 |
| Sex |  |  |  |
| Female | reference |  |  |
| Male | 0.98 | 0.60 | 1.59 |
| Race/ethnicity |  |  |  |
| White | reference |  |  |
| Non-white | 0.95 | 0.66 | 1.36 |
| PE Severity Index Class |  |  |  |
| I | reference |  |  |
| II | 4.33 | 1.62 | 11.57 |
| III | 3.66 | 1.31 | 10.23 |
| IV | 7.75 | 3.01 | 19.96 |
| V | 13.55 | 4.08 | 45.03 |
| (Pre)syncope Classification |  |  |  |
| Neither | reference |  |  |
| Presyncope | 2.68 | 1.37 | 5.25 |
| Syncope | 4.61 | 2.54 | 8.38 |
| Ventilatory Support* |  |  |  |
| None | reference |  |  |
| Any | 4.57 | 2.87 | 7.29 |
| Clot Location on CTPA† |  |  |  |
| Distal | reference |  |  |
| Proximal | 2.30 | 1.46 | 3.61 |
| Unclear or not measured | 0.72 | 0.29 | 1.83 |
| Submassive PE‡ |  |  |  |
| No | reference |  |  |
| Yes | 3.29 | 2.38 | 4.56 |

*CI,* confidence interval*; CTPA,* computed tomography pulmonary angiography; *PE*, pulmonary embolism.

* Includes non-rebreather mask, non-invasive ventilation, and endotracheal intubation with mechanical ventilation.

†Proximal emboli were clearly lobar or more proximal, whereas distal emboli were “segmental or lobar” or more distal. Location was not measured in patients whose PE was diagnosed with ventilation/perfusion scan.

‡ Submassive PE were not hypotensive (per massive criteria), yet had an elevated ED troponin level, an elevated B-type natriuretic peptide level, or right ventricular dysfunction on echocardiogram.

**Table S2**. Association between patient characteristics and 30-day all-cause mortality of emergency department patients with non-massive acute pulmonary embolism (n=2,969).

|  | Multivariate Model | | |
| --- | --- | --- | --- |
|  | Adjusted Odds Ratio | 95% CI | |
| Age, per year | 1.00 | 0.99 | 1.01 |
| Sex |  |  |  |
| Female | reference |  |  |
| Male | 0.67 | 0.46 | 0.99 |
| Race/ethnicity |  |  |  |
| White | reference |  |  |
| Non-white | 1.04 | 0.65 | 1.67 |
| PE Severity Index Class |  |  |  |
| I-II | reference |  |  |
| III-V | 27.03 | 9.76 | 74.88 |
| (Pre)syncope Categorization |  |  |  |
| Neither |  |  |  |
| Presyncope | 0.77 | 0.24 | 2.49 |
| Syncope | 1.78 | 0.72 | 4.40 |
| Ventilatory Support* |  |  |  |
| None | reference |  |  |
| Any | 3.76 | 2.75 | 5.14 |
| Clot Location on CTPA^†^ |  |  |  |
| Distal | reference |  |  |
| Proximal | 0.96 | 0.68 | 1.38 |
| Unclear or not measured | 1.52 | 0.78 | 2.96 |
| Submassive PE^‡^ |  |  |  |
| No | reference |  |  |
| Yes | 1.03 | 0.75 | 1.41 |

*CTPA,* computed tomography pulmonary angiography; *PE*, pulmonary embolism.

* Includes non-rebreather mask, non-invasive ventilation, and endotracheal intubation with mechanical ventilation.

†Proximal emboli were clearly lobar or more proximal, whereas distal emboli were “segmental or lobar” or more distal.

‡ Submassive PE were not hypotensive (per massive criteria), yet had an elevated ED troponin level, an elevated B-type natriuretic peptide, or right ventricular dysfunction on echocardiogram.
